# Alcohol’s Effects on Lung Health and Immunity

**DOI:** 10.35946/arcr.v37.2.05

**Published:** 2015

**Authors:** Samantha M. Simet, Joseph H. Sisson

**Affiliations:** Samantha M. Simet, Ph.D., is a postdoctoral fellow, and Joseph H. Sisson, M.D., is Larson Professor and division chief in the Pulmonary, Critical Care, Sleep & Allergy Division, University of Nebraska Medical Center, Omaha, Nebraska.

**Keywords:** Alcohol use, abuse, and dependence, alcohol use disorder, immunity, impaired immune response, innate immune response, lung disorders, pneumonia, tuberculosis, respiratory syncytial virus infection, acute respiratory distress syndrome, pulmonary infection, neutrophils, lymphocytes, alveolar macrophages, pathophysiology

## Abstract

It has long been known that people with alcohol use disorder (AUD) not only may develop physical dependence but also may experience devastating long-term health problems. The most common and identifiable alcohol-associated health problems include liver cirrhosis, pancreatitis, cardiomyopathies, neuropathies, and dementia. However, the lung also is adversely affected by alcohol abuse, a fact often overlooked by clinicians and the public. Individuals with AUD are more likely to develop pneumonia, tuberculosis (TB), respiratory syncytial virus (RSV) infection, and acute respiratory distress syndrome (ARDS). Increased susceptibility to these and other pulmonary infections is caused by impaired immune responses in people with AUD. The key immune cells involved in combating pulmonary conditions such as pneumonia, TB, RSV infection, and ARDS are neutrophils, lymphocytes, alveolar macrophages, and the cells responsible for innate immune responses. Researchers are only now beginning to understand how alcohol affects these cells and how these effects contribute to the pathophysiology of pulmonary diseases in people with AUD.

People have been drinking alcoholic beverages for millennia, and alcohol consumption has played an important role throughout human history, being linked to ancient and modern religions, early medicine, and social occasions and celebrations. Although alcohol consumption is socially accepted across many cultures, heavy and prolonged alcohol intake can lead not only to physical dependence but also to devastating long-term health problems. An estimated 18 million Americans have alcohol use disorder (AUD), including alcoholism and harmful drinking (National Institute on Alcohol Abuse and Alcoholism [NIAAA] 2014). NIAAA (2014) has established guidelines for low-risk drinking that are age and gender specific. Thus, for men ages 21–64, low-risk drinking is defined as consumption of no more than 4 drinks per day or 14 drinks per week. For women, as well as for men ages 65 and older, drinking levels for low-risk drinking are defined as no more than 3 drinks per occasion or 7 drinks per week. Exceeding these daily or weekly drinking limits significantly increases the risk of developing AUD and problematic health outcomes (NIAAA 2014).

The most common health problems associated with AUD are liver cirrhosis, pancreatitis, damage to the heart muscles (i.e., cardiomyopathies), nerve damage (i.e., neuropathies), and dementia (Lieber 1995). However, the lung also is adversely affected by alcohol abuse, a fact that often is overlooked by clinicians and the public. For example, it is clear that heavy drinkers are more likely to have pneumonia (Jellinek 1943; Samokhvalov et al. 2010), tuberculosis (TB) (Borgdorff et al. 1998; Buskin et al. 1994; Kline et al. 1995; Narasimhan et al. 2013), respiratory syncytial virus (RSV) infection (Jerrells et al. 2007), and acute respiratory distress syndrome (ARDS) (Moore et al. 2003; Moss et al. 1996). In recent years, researchers have come to better understand the pathophysiology of lung injury in individuals with AUD and the role that alcohol’s effects on lung immune responses play in this process. This review focuses on these four common pulmonary conditions associated with AUD and their pathophysiologic lung immune responses.

## Bacterial Pneumonia

One of the most common and deadliest conditions afflicting individuals with AUD is bacterial pneumonia. Dr. Benjamin Rush, the first Surgeon General of the United States, described some of the earliest links of alcohol abuse to pneumonia over two centuries ago, reporting that pneumonia was more common in drinkers than nondrinkers (Jellinek 1943; Rush 1810). Two centuries later, the correlation between alcohol abuse and lung infections still remains strong. According to the Centers for Disease Control and Prevention (CDC), people who abuse alcohol are 10 times more likely to develop pneumococcal pneumonia and 4 times more likely to die from pneumonia than nondrinkers (Lujan et al. 2010).

Pneumococcal pneumonia, caused by the bacterium *Streptococcus pneumoniae,* is the most common type of pneumonia in both healthy individuals and heavy alcohol users (Ruiz et al. 1999). In addition, the incidence of infections with *Klebsiella pneumoniae* also is increased in people with AUD and seems to cause disproportionate rates of lung infection and high mortality in this population (Feldman et al. 1990; Limson et al. 1956). Regardless of the bacterial pathogen causing the infection, dysfunction of the host’s immune responses to bacterial pneumonia, particularly those involving macrophages in the lungs (i.e., alveolar macrophages) and neutrophils, is an important contributor to the pathogenesis of the disease in people with AUD. The alveolar macrophages eliminate pathogens by ingesting them—a process known as phagocytosis—whereas neutrophils are involved in inflammatory responses.

Alveolar macrophages are the first line of defense in lung cellular immunity. These phagocytic cells ingest and clear inhaled microbes and foreign particles from the lungs. The release of cytokines and chemokines by these cells, in turn, mediates the influx of neutrophils into the lungs that occurs in response to infection. Chronic alcohol exposure significantly interferes with alveolar macrophage function. Prolonged alcohol consumption impairs the cells’ phagocytic capacity (Joshi et al. 2005, 2009), release of cytokines and chemokines (D’Souza et al. 1996), and release of neutrophil chemoattractants (Craig et al. 2009). Although alveolar macrophages are the primary residential innate immune cells and play a pivotal role in the clearance of bacterial and viral pathogens, understanding of and research on their specific function in the context of heavy alcohol consumption and AUD still is lacking. It is clear, however, that prolonged alcohol consumption alters the pathophysiology and key factors involved in neutrophil-driven lung immunity in response to *S. pneumoniae* infection. Thus, studies have shown that exposure to alcohol impairs neutrophil recruitment (Gluckman and MacGregor 1978), weakens phagocytosis of pathogens by neutrophils (Boe et al. 2001; Jareo et al. 1995), and reduces neutrophil production and release of neutrophils into circulating blood (Melvan et al. 2011; Siggins et al. 2011). The following paragraphs outline the data supporting these deleterious effects of heavy alcohol consumption on neutrophil function in the context of *S. pneumoniae* lung infections.

Neutrophils are the earliest immune effector cells recruited to the site of inflammation during a bacteria-triggered inflammatory response. In the case of pneumonia, neutrophil recruitment to the lung is a critical early step in the host’s immune response. In the early stages of infection, circulating neutrophils are recruited to sites of inflammation by a gradient of inflammatory mediators, including proinflammatory cytokines and chemokines. Neutrophils traverse the cells lining the blood vessels (i.e., vasculature endothelial cells) into the space between the lung cells (i.e., the interstitial space of the lung). From there, they migrate into the airspace within the alveoli to the sites of microbial invasion. Once in the alveolar space, neutrophils ingest, degrade, and remove invading pathogens (Nathan 2006). This neutrophil-recruitment process is impaired by alcohol; even brief alcohol exposure decreases neutrophil recruitment to infected sites (Astry et al. 1983). For example, alcohol studies in rodents infected with aerosolized *Staphylococcus aureus* or *Proteus mirabilis* have demonstrated that alcohol intoxication decreases bacterial clearance in conjunction with decreased pulmonary neutrophil recruitment (Astry et al. 1983). Similarly, Boe and colleagues (2001) found that alcohol-exposed rats had decreased pulmonary neutrophil recruitment for up to 18 hours following *S. pneumoniae* challenge; after that, however, neutrophil recruitment remained elevated even 40 hours post-challenge compared with nondrinking rats. This observation suggests that in individuals with heavy alcohol exposure, the host neutrophils arrive late at the infected lung but stay longer (Sisson et al. 2005). Impaired neutrophil recruitment also has been reported in human volunteers with blood alcohol concentrations (BACs) of 0.10 percent and 0.24 percent (Gluckman and MacGregor 1978)—that is, even at BACs that only slightly exceed the threshold for legal intoxication in the United States (i.e., 0.08 percent). These findings highlight that alcohol intoxication impairs neutrophil recruitment into infected tissues and the lung and also hinders neutrophil clearance from the lung.

The alcohol-induced dysregulation of lung neutrophil recruitment and clearance is only part of the problem in people with AUD, because alcohol also has harmful effects on other aspects of neutrophil functioning. However, alcohol’s effects on neutrophil phagocytosis and pathogen killing are less clear than the effects on neutrophil recruitment, and the findings to date are inconclusive. Thus, some studies indicate that alcohol has no effect on neutrophil phagocytosis or pathogen killing (Nilsson et al. 1996; Spagnuolo and MacGregor 1975), whereas other studies demonstrate that acute alcohol exposure impairs functional activities of neutrophils. For example, Davis and colleagues (1991) found that alcohol-fed rats failed to clear bacteria from the lungs and had increased mortality. Some of this discrepancy likely is related to differences in the bacterial pathogens studied. Thus, Jareo and colleagues (1995) noted impaired neutrophil killing of selected strains of *S. pneumoniae* in vitro and a complete absence of killing of other bacterial strains in alcohol-exposed animals. In human studies, BACs as low as 0.2 percent (i.e., approximately 2.5 times the legal intoxication level) impaired neutrophil degranulation and bactericidal activity (Tamura et al. 1998).

In addition to neutrophil recruitment to infected areas and reduced neutrophil-killing potential, production of these cells also is affected. In healthy individuals, the bone marrow produces approximately 120 billion neutrophils per day (Cartwright et al. 1964; von Vietinghoff and Ley 2008). Moreover, bone-marrow neutrophil production is significantly increased 24 to 48 hours after a systemic bacterial infection (Melvan et al. 2011). Several studies observed decreased numbers of neutrophils in people with AUD. Alcohol exposure suppresses neutrophil production by the bone marrow and other blood cell–producing (i.e., hematopoietic) tissues (Melvan et al. 2011; Raasch et al. 2010; Siggins et al. 2011). This decreased neutrophil proliferation may account for the decreased number of neutrophils found in the lungs during the host response to pneumonia following alcohol consumption. Alcohol primarily suppresses neutrophil production by interfering with the actions of granulocyte colony-stimulating factor (G-CSF), which is the principal driver of neutrophil production, maturation, and function in the bone marrow and inflamed tissues (Bagby et al. 1998). G-CSF levels normally increase in situations where more neutrophils are needed. Thus, G-CSF levels rise significantly within 3 hours of pulmonary bacterial infections, peaking at 12 hours, and plateauing around 18 hours post-infection within the lung and systemic circulation. Additional studies have demonstrated that alcohol-consuming animals are more likely to succumb to *S. pneumoniae* within 2 to 4 days following infection compared with their nondrinking counterparts (Boe et al. 2001). Alcohol-induced suppression of G-CSF–driven neutrophil production combined with impaired bacterial clearance likely account for the high severity and mortality of bacterial infections among the alcohol-fed mice observed in these studies.

Because of the key role of G-CSF in neutrophil regulation, investigators have hypothesized that alcohol-induced neutrophil dysfunction can be prevented by pretreatment with G-CSF (Nelson et al. 1991). Indeed, pre-treatment of alcohol-consuming mice with G-CSF for 2 days before *K. pneumoniae* infection increased neutrophil recruitment compared with that of control animals not receiving G-CSF. In addition to increased neutrophil recruitment, the pre-treated animals also exhibited improved bacterial killing and decreased mortality (Nelson et al. 1991). The findings indicate that G-CSF can prevent alcohol-induced deficits in neutrophil-dependent pulmonary defenses by increasing neutrophil production and bacterial killing function.

In summary, in the context of lung bacterial infections, alcohol impairs neutrophil recruitment (Gluckman and MacGregor 1978), reduces pathogen killing through phagocytosis (Boe et al. 2001; Jareo et al. 1995), and decreases neutrophil production and release of neutrophils into circulating blood (Melvan et al. 2011; Siggins et al. 2011). Pretreatment with G-CSF ameliorates alcohol-induced neutrophil dysfunction, including impairments in neutrophil recruitment and bacterial killing.

## Tuberculosis

Bacterial pneumonia is not the only infectious disease with an increased risk among people with AUD. Lung infections with *Mycobacterium tuberculosis*, the underlying pathogen of TB, also occur at higher rates in this population (Jellinek 1943; World Health Organization [WHO] 2014). TB is the second-leading cause of death worldwide, accounting for 1.3 million deaths in 2012. The disease is spread from person to person through the air, when infected people cough, sneeze, speak, or sing, thereby releasing *M. tuberculosis* into the air (WHO 2014). Interestingly, not everyone infected with *M. tuberculosis* becomes sick. The infection can remain latent for years while the host’s immune system is able to combat it. The infected individual will have no symptoms and is not infectious to others. However, latent TB may become active when the immune system is weakened. Alcohol abuse is therefore a risk factor for active TB (Borgdorff et al. 1998; Buskin et al. 1994; Kline et al. 1995; Narasimhan et al. 2013).

Although TB is treatable with antibiotics, the prevalence of multidrug-resistant tuberculosis (MDRTB) is on the rise and has been reported worldwide (WHO 2014). One of the main factors increasing the prevalence of MDRTB is noncompliance by patients who do not complete their normal 6-month treatment regimen, leading to the emergence of drug-resistant *M. tuberculosis*. A recent study of MDRTB in South Africa reports that of 225 patients diagnosed with MDRTB, only 50 percent were cured or completed treatment. Treatment default rates were highest among alcohol users (Kendall et al. 2013). Other countries also report similar TB treatment defaults in individuals with AUD, resulting in poorer treatment outcomes and increased mortality rates (Bumburidi et al. 2006; Jakubowiak et al. 2007). Along with noncompliance, people with AUD have compromised lymphocytes, which are among the main immune components combating TB infections. The three main types of lymphocytes are natural killer (NK) cells, T cells, and B cells. Chronic alcohol intake modulates the functions of all three of these lymphocyte populations (Cook 1998; Lundy et al. 1975; Meadows et al. 1992; Spinozzi et al. 1992; Szabo 1999).

NK cells do not need previous exposure to their target cells to recognize, bind to, and destroy these targets (e.g., cancer and virus-infected cells) (Vivier et al. 2008). In a mouse model, NK cells also become activated during the early response to *M. tuberculosis* infection and produce interferon γ (INF-γ), an important cytokine that stimulates cell-mediated immunity (Junqueira-Kipnis et al. 2003). Alcohol consumption in mice reduces the in vitro killing capacity of NK cells compared with control animals not exposed to alcohol (Meadows et al. 1992).

Chronic alcohol intake impairs not only the killing capacity of NK cells but also diminishes normal functioning of various types of T cells, which primarily mediate the immune response to TB (Gambon-Deza et al. 1995). (For more information on the types of T cells, see the textbox.) Alcohol exposure affects T-cell function through a variety of pathways:

- People with AUD often have reduced numbers of lymphocytes (i.e., lymphopenia), alterations in the T-cell compartments (Cook 1998; Szabo 1999; Tonnesen et al. 1990), decreased response to substances that stimulate cell division (i.e., mitogen-stimulation response) (Spinozzi et al. 1991), and impaired delayed-type hypersensitive responses (Lundy et al. 1975).^1^
- Chronic alcohol consumption interferes with the proper presentation of pathogen-derived molecules (i.e., antigens), which is required for T- and B-cell activation (Ness et al. 2008).
- Alcohol-exposed T cells have a reduced capacity to produce IFN-γ compared with control cells (Chadha et al. 1991).
- Alcohol-fed mice infected with TB exhibit decreased numbers of the two main subtypes of T cells (i.e., CD4^+^ and CD8^+^ T cells) as well as decreased proliferation of these cells compared with control mice (Mason et al. 2004).

IFN-γ–producing (i.e., type 1) T cells mediate immune reactions that are responsible for fighting not only *M. tuberculosis* infections but also infections by other bacterial pathogens, such as *K. pneumoniae* (Greenberger et al. 1996; Moore et al. 2002). Infection with *K. pneumoniae* induces time-dependent release of IL-12 from T cells, which in turn drives T cell IFN-γ production. This chain of reactions is disrupted by alcohol, because the levels of both IL-12 and IFN-γ were decreased in alcohol-exposed mice infected with *K. pneumoniae* (Zisman et al. 1998). These deficits could account for decreased clearance of these bacteria from the lungs. In addition to this flawed type-1 (Th1) response, the lungs of alcohol-fed rodents exhibit increased amounts of the inflammatory cytokine IL-10, which also may contribute to impaired lung clearance because normalizing IL-10 levels within the pulmonary system improves bacterial lung clearance (Greenberger et al. 1995).

**Types of T Cells**

T cells are an important part of the immune system and fulfill a variety of functions in defending the organism against various pathogens. To do this, T cells are divided into different subgroups that all have specific functions. The two main subgroups are T helper cells and cytotoxic T cells. T helper cells, as the name implies, assist other immune cells in various ways. These T cells are characterized by the presence of a molecule called CD4 on their surface and therefore also are called CD4^+^ cells. When they become activated, CD4^+^ cells secrete various cytokines to facilitate different types of immune responses. Depending on the exact cytokines they produce, they can be further classified. For example, type 1 CD4^+^ cells are characterized by the secretion of interferon γ (IFN-γ); they act primarily against pathogens that are found within cells. Conversely, type 2 CD4^+^ cells do not produce IFN-γ but various types of interleukins. These cells act primarily against pathogens that are found outside the cells.

The other main subgroup of T cells, the cytotoxic T cells, has CD8 molecules on their surfaces. They are therefore also known as CD8^+^ cells. These T cells directly destroy virus-infected and tumor cells.

B cells are responsible for the second arm of the immune response (i.e., the humoral immunity) that is mediated not by specific cells but by immune molecules (i.e., antibodies) produced and secreted by B cells in response to exposure to a pathogen. These antibodies consist of molecules called immunoglobulins (Igs). There are different types of Igs (e.g., IgA, IgM, and IgG) that all have specific functions during the immune response. Alcohol exposure in the context of TB also affects this arm of the immune response. Thus, although the total number of circulating B cells does not differ significantly between people with and without AUD, people with AUD have elevated levels of circulating IgA, IgM, and IgG (Spinozzi et al. 1992). In the lungs of people with AUD, however, Ig levels are reduced as determined by bronchoalveolar lavage (BAL) (Spinozzi et al. 1992). Replacement IgG therapy only partially restored Ig levels in these people, although it decreased the rates of pulmonary infections (Spinozzi et al. 1992).

## RSV Infection

Although much of the attention concerning lung infections in people with AUD has been focused on bacterial infections, these individuals also have an increased susceptibility to viral airway infections. RSV is one of the most common lower respiratory tract viral pathogens and is a major cause of respiratory infections in children. Although RSV infections once were thought to be limited to children, it is now clear that RSV also is a serious problem in older people, patients with chronic obstructive pulmonary disease (COPD), and people with AUD. Prolonged alcohol exposure alters the first line of the innate cellular defense, the mucociliary apparatus, against invading pathogens such as RSV. This defense system propels inhaled particles, microbes, toxins, and debris out of the lungs and airways with the help of the fine hairs (i.e., cilia) on the cells that line the respiratory tract.

Alcohol has unique effects on the ciliated airways because it is rapidly and transiently absorbed from the bronchial circulation directly across the ciliated epithelium of the conducting airways. It then is vaporized into the airways and excreted during exhalation. However, when the exhaled air cools as it reaches the trachea, the alcohol vapor condenses and is dissolved back into the fluid in periciliary airway lining (George et al. 1996). This recycling of alcohol vapor continually subjects the conducting airways to high concentrations of alcohol (George et al. 1996), which modify airway-epithelium host defenses by altering cytokine release, barrier function (Simet et al. 2012), and cilia function (Sisson 1995; Sisson et al. 2009; Wyatt and Sisson 2001).

As is the case with other organs, alcohol’s specific effects on the conducting airways depend on the route, dose, and length of the exposure (Sisson 2007). Early studies found that direct exposure of the ciliated airways to very high and nonbiologically relevant alcohol concentrations (i.e., 4 to 10 percent or 0.8–3.2 M) interfere with the movement of the cilia (i.e., cause ciliostasis) in a concentration-dependent manner (Nungester and Klepser 1938; Purkinjie and Valentine 1835). More recent studies have established that biologically relevant alcohol concentrations have very focused and specific effects on the lung airways. Over the past two decades, studies demonstrated that brief exposure to modest alcohol concentrations triggers generation of nitric oxide (NO) in the airway epithelial cells. This NO production stimulates a signaling pathway that involves the enzyme guanylyl cyclase, which produces a compound called cyclic guanosine monophosphate (cGMP). cGMP, in turn, activates cGMP-dependent protein kinase (PKG), followed by activation of the cyclic adenosine monophosphate (cAMP)-dependent protein kinase A (PKA). Activation of this dual kinase signaling pathway results in faster cilia beat frequency (CBF) in cilia briefly exposed to a moderate alcohol dose compared with controls (Sisson 1995; Sisson et al. 2009; Stout et al. 2007; Wyatt et al. 2003). More recent studies demonstrated that this rapid and transient alcohol-induced increase in NO levels was triggered by the alcohol-induced phosphorylation of heat shock protein 90 (HSP90) (Simet et al. 2013*b*). Upon phosphorylation, HSP90 increases its association with endothelial nitric oxide synthase (eNOS) in cilia, which then activates the cyclase–kinase cascade, resulting in increased CBF (Simet et al. 2013*b*). These findings are counterintuitive to the conventional wisdom that alcohol interferes with lung host defenses because stimulation of CBF should protect the lung; however, the clinical observation is that heavy alcohol exposure impairs lung host defenses. Indeed, that is just the first part of the story.

In contrast to brief alcohol exposure, prolonged alcohol exposure completely desensitizes lung airway cilia such that they can no longer beat faster when exposed to inhaled pathogens. This cilia-desensitization effect is known as alcohol-induced cilia dysfunction (AICD). In AICD, prolonged alcohol exposure results in failure to stimulate CBF, thereby desensitizing cilia to activating agents such as beta agonists (Wyatt and Sisson 2001). AICD likely results from decreased HSP90/eNOS association, which in turn attenuates the NO-stimulated cGMP/cAMP-dependent kinase activation pathway (Simet et al. 2013*a*; Wyatt and Sisson 2001). Alternatively, AICD may be related to oxidant-driven eNOS uncoupling, because AICD can be prevented in alcohol-drinking mice by concurrently feeding the animals dietary antioxidants, such as Procysteine™ or N-acetylcysteine (Simet et al. 2013*a*).

Regardless of the exact underlying mechanism, the consequence of alcohol-induced impairment in airway ciliary function is increased susceptibility to airway bacterial and viral infections, such as RSV. For example, Jerrells and colleagues (2007) demonstrated that alcohol-fed mice are inefficient in clearing RSV from the lungs. In addition, the alcohol-consuming mice exhibited enhanced and prolonged RSV infection compared with nondrinking RSV-infected animals. RSV infection itself causes a significant loss of ciliated cells from the airway epithelium and the remaining cilia beat more slowly compared with control cells from uninfected epithelia (Slager et al. 2006). This ciliary slowing is regulated by the activation of another signaling protein called protein kinase Cɛ (PKCɛ); moreover, once PKCɛ becomes inactivated again, the ciliated cells detach from the epithelium (Slager et al. 2006). It is unknown how concurrent alcohol exposure impacts these consequences of RSV infection. In summary, these studies demonstrate that alcohol exposure compromises innate defenses against viral pathogens such as RSV in part by disrupting airway ciliary function.

## ARDS

People with AUD who experience any type of lung injury—be it caused by infections with bacteria, TB-causing *M. tuberculosis*, or viruses or by noninfectious events such as trauma, pancreatitis, or burns—are at high risk for developing ARDS. The syndrome is characterized by endothelial and alveolar epithelial barrier dysfunction, severe inflammation, and surfactant dysfunction.^2^ During ARDS, robust lung inflammation results in increased accumulation of fluid and inflammatory cells in the alveolar spaces. This causes impaired gas exchange in the lung, resulting in decreased oxygenation of the blood and multiple organ failure caused by the insufficient oxygen levels. ARDS is a life-threatening complication that develops in response to several events, including lung infection, non-lung sepsis, aspiration of stomach contents, trauma, and/or inhaled toxins. Among the most common causes of ARDS are bacterial pneumonia and an associated severe inflammatory response (i.e., alveolar sepsis). Alcohol abuse also has been identified as an independent risk factor that increases the odds of at-risk individuals to develop ARDS (Moss et al. 1996). Indeed, ARDS is two to four times more common in individuals with AUD than in non-AUD individuals (Moss and Burnham 2003).

One of the central features of ARDS is an impaired barrier function of the alveolar epithelial and endothelial cells.^3^ Studies on the effect of alcohol alone on alveolar barrier function have revealed that chronic alcohol intake alters physical barrier properties within alveoli (Guidot et al. 2000). Interestingly, alveolar cells from ethanol-fed rats had increased expression of sodium channels in the membrane facing the interior of the alveoli (i.e., the apical membrane). This up-regulation of sodium channels may counteract the increased paracellular leak from the blood space into the alveolar airspace observed in the lungs of alcoholic subjects, and may explain why prolonged alcohol intake, in the absence of inflammation, does not result in fluid accumulation in the lungs (i.e., pulmonary edema) (Guidot and Hart 2005). However, these alcohol-fed rats had diminished airway clearance when challenged with saline, even in the absence of an inflammatory challenge (Guidot et al. 2000). These data suggest that the alveolar epithelium actually is dysfunctional after alcohol exposure, even though it seems normal and is able to regulate the normal air–liquid interface by enhancing sodium channels at the apical surface. In the presence of an inflammatory reaction, the compensatory mechanism likely becomes overwhelmed, resulting in greater susceptibility to barrier disruption and flooding of the alveolar space with protein-containing fluid.

One of the molecules involved in disrupting epithelial integrity is the cytokine transforming growth factor β_1_ (TGF-β_1_). Studies in rats that had been fed alcohol for a prolonged period of time found that expression of inactive TGF-β_1_ protein doubled in lung tissue compared with nondrinking animals; however, there was no evidence of TGF-β_1_ release or activation in the absence of an infection (Bechara et al. 2004). Nevertheless, alcohol-fed rats released five times more activated TGF-β_1_ into the alveolar airspaces than did nondrinking rats in the presence of bacterial toxins in their blood (i.e., during endotoxemia). Additional studies using alveolar epithelial cell layers derived from these alcohol-fed rats found that this permeability defect was inhibited by neutralizing antibodies to TGF-β_1_ (Bechara et al. 2004). Together, these data suggest that prolonged alcohol intake increases TGF-β_1_ levels, which during inflammatory responses can be released and activated in the alveolar space, where it can directly impair epithelial barrier properties (Guidot and Hart 2005).

Another fundamental component contributing to alcohol’s effects on the lungs is oxidative stress and the resulting alterations in alveolar macrophage function. As mentioned previously, alveolar macrophages are key components of both innate and acquired immunity against invading pathogens in the lung. After mucociliary clearance, these cells are the next line of cellular defense against invading pathogens through their phagocytic, microbiocidal, and secretory functions (Rubins 2003). Chronic alcohol ingestion decreases alveolar macrophage function by inhibiting the release of cytokines and chemokines as well as other factors essential for microbial killing and immune response (Franke-Ullmann et al. 1996; Omidvari et al. 1998). Alcohol-induced alveolar macrophage dysfunction likely occurs primarily as a result of alcohol-induced increases in oxidative stress, which is reflected by depletion of the antioxidant glutathione (GSH) in BAL fluid (Brown et al. 2007; Yeh et al. 2007). Impaired secretion of granulocyte monocyte colony-stimulating factor (GM-CSF) by type II alveolar cells likely also contributes to alcohol-induced oxidative stress (Joshi et al. 2005).

The alcohol-associated oxidative stress in the lungs is related at least in part to alcohol-driven changes in NADPH oxidase (Nox) enzyme function and GSH depletion. Nox enzymes generally promote oxidative stress, whereas antioxidants such as GSH help protect the cells against oxidative stress. Increased levels of Nox enzymes (e.g., Nox_4_) and decreased GSH pools are emerging as significant components of the processes through which alcohol induces oxidative stress that then causes alveolar macrophage dysfunction. As mentioned previously, chronic alcohol intake increases the levels of activated TGF-β_1_, which then upregulates and activates Nox_4_ (Brown and Griendling 2009). Nox_4_ activation in turn leads to activation of Nox_1_ and Nox_2_, both of which cause production of reactive oxygen species (ROS) in the alveolar macrophages (Yeligar et al. 2012). At the same time, chronic alcohol consumption depletes levels of GSH in the lungs. Both of these processes promote chronic oxidative stress, which then impairs alveolar macrophage functions (Brown et al. 2004, 2007; Holguin et al. 1998; Yeh et al. 2007). Thus, both cellular-based microbial lung clearance and alveolar macrophage cell viability are decreased after chronic alcohol exposure and the resulting increase in oxidative stress (Velasquez et al. 2002). This role of alcohol-induced oxidative stress in macrophage dysfunction has been demonstrated in animal models in which chronic alcohol-drinking mice had decreased levels of GSH and increased levels of Nox enzymes and Nox-associated proteins in alveolar macrophages (Yeligar et al. 2012, 2014).

The identification of alcohol-driven oxidative stress as a contributor to alveolar macrophage dysfunction has led to promising antioxidant treatment approaches aiming to prevent alcohol-induced lung conditions in rodent models of prolonged alcohol consumption. For example, oral GSH treatment in alcohol-drinking mice was able to restore GSH pools, reverse alcohol-induced Nox increases, and restore alveolar macrophage function (Yeligar et al. 2012, 2014). Other studies have demonstrated that treatment with GSH precursors such as Procysteine™, N-acetylcysteine, or s-adenosylmethionine was able to improve alveolar macrophage phagocytosis (Brown et al. 2007) and promote differentiation of interstitial macrophages into mature alveolar macrophages (Brown et al. 2009) during chronic alcohol ingestion. These results suggest that GSH is a vital component in restoring alcohol-induced alveolar macrophage function by decreasing Nox proteins and restoring GSH pools.

Studies also have analyzed the role of GM-CSF in alcohol-induced oxidative stress and impaired lung immunity. GM-CSF is secreted by type II alveolar cells and is required for terminal differentiation of circulating monocytes into mature, functional alveolar macrophages (Joshi et al. 2006). The levels of GM-CSF are reduced in chronic alcohol-drinking mice (Joshi et al. 2005). Studies have shown that mice that have been genetically modified to no longer produce GM-CSF (i.e., GM-CSF knockout mice) exhibit a variety of changes contributing to impaired lung immune responses, including impaired surfactant expression, clearance, and phagocytosis; decreased expression of GM-CSF receptor; and impaired alveolar macrophage development (Dranoff et al. 1994; Joshi et al. 2005; Trapnell and Whitsett 2002). Conversely, overexpression of GM-CSF in genetically modified (i.e., transgenic) mice causes increased lung size, excessive growth (i.e., hyperplasia) of alveolar epithelial cells, and improved surfactant protein removal from the alveolar space (Ikegami et al. 1997). Other studies using a rat model of chronic alcohol consumption found that although the levels of GM-CSF in the alveolar space were not affected by alcohol exposure, the expression of GM-CSF receptors was significantly decreased in the membranes of alveolar macrophages (Joshi et al. 2005). Chronic alcohol intake also decreased alveolar binding of PU.1, a transcription factor responsible for GM-CSF activation. When the animals were treated with recombinant GM-CSF, alveolar macrophage bacterial phagocytic capacity, GM-CSF receptor expression, and PU.1 nuclear binding were restored (Joshi et al. 2005). These studies offer the groundwork for understanding the importance of GM-CSF within the lung for the maturation and host immune function of the alveolar macrophage as well as the deleterious impact of chronic alcohol use on these processes.

As these experimental studies have demonstrated, chronic alcohol intake exerts a detrimental effect on the function of alveolar macrophages, an important cell type involved in limiting ARDS risk and severity. Restoration of GM-CSF following alcohol exposure, replenishing of GSH pools, and normalization of Nox enzymes restore alveolar macrophage functions. The use of recombinant GM-CSF and antioxidants potentially could improve alveolar macrophage function in people with AUD. Preventing the pathophysiological consequences of lung injury, including excessive inflammation, and the resulting pulmonary edema and insufficient oxygen supply (i.e., hypoxia) in the tissues associated with ARDS remains the goal of research on alcohol-enhanced ARDS.

## Summary

For centuries, it has been known that people with AUD are more likely to have pulmonary infections such as pneumonia and TB. Over the past two decades, it has become clear that other conditions such as RSV and ARDS also are linked to high-risk alcohol consumption. Even with the development of antibiotics, vaccinations, health education, and preventative medicine, a strong correlation still exists among heavy alcohol consumption, pulmonary infections, and ARDS. Over the past 30 years, however, research has vastly enhanced our understanding of the pathophysiology of the immunocompromised “alcoholic lung.” This includes new insight into the mechanisms that cause the harmful effects of heavy alcohol intake on neutrophils, lymphocytes, airway ciliary function, and alveolar macrophages, all of which contribute to the prolonged and often more severe pulmonary diseases observed in people who abuse alcohol. Armed with a better understanding of the lung pathophysiology unique to the heavy drinker, clinicians now are better prepared to combat these diseases through various treatment regimens. Preclinical models suggest that antioxidant nutritional supplements may prevent alcohol-induced lung oxidative stress, allowing mucociliary clearance and alveolar macrophage functions to be preserved. Promising animal studies also show that restoration of normal G-CSF, IgG, and GM-CSF levels could permit normal lung recovery following infection and injury in individuals with AUD. These disease- and cell-associated studies offer hope for novel preventative and therapeutic options for restoration of a normal lung immune response in people with AUD.

## Footnotes

1 Delayed-type hypersensitivity responses are excessive immune reactions that occur only a few days after the body has been exposed to the pathogen. These responses are not mediated by immune molecules produced by B cells (i.e., antibodies) but by T cells.

2 Surfactant is a lipoprotein complex produced by alveolar cells that covers alveoli and helps ensure proper lung function.

3 The epithelial cells line the alveolar surface that faces the inside (or airspace) of alveoli, whereas the endothelial cells line the surface that faces the outside of the alveoli and the surrounding blood vessels.
